# Transcriptional Regulation of Carbohydrate Utilization Pathways in the *Bifidobacterium* Genus

**DOI:** 10.3389/fmicb.2016.00120

**Published:** 2016-02-09

**Authors:** Matvei S. Khoroshkin, Semen A. Leyn, Douwe Van Sinderen, Dmitry A. Rodionov

**Affiliations:** ^1^A. A. Kharkevich Institute for Information Transmission Problems, Russian Academy of SciencesMoscow, Russia; ^2^School of Microbiology and Alimentary Pharmabiotic Centre Microbiome Institute, University College CorkCork, Ireland; ^3^Sanford Burnham Prebys Medical Discovery InstituteLa Jolla, CA, USA

**Keywords:** carbohydrate metabolsim, sugar catabolism, transcription factors, regulatory networks, regulatory sequences, nucleic acid, bifidobacteria, comparative genomics

## Abstract

Bifidobacteria, which represent common commensals of mammalian gut, are believed to have positive effects on human health. The influence of certain non-digestible carbohydrates (and their use as so-called prebiotics) on growth and metabolic activity of bifidobacteria is of increasing interest; however, mechanisms of transcriptional control of carbohydrate metabolism are poorly understood in these species. We used a comparative genomics approach to reconstruct carbohydrate utilization pathways and transcriptional regulons in 10 *Bifidobacterium* genomes. Analysis of regulatory gene regions revealed candidate DNA motifs and reconstructed regulons for 268 transcription factors from the LacI, ROK, DeoR, AraC, GntR, and TetR families that form 64 orthologous groups of regulators. Most of the reconstructed regulons are local and control specific catabolic pathways for host- and diet-derived glycans and monosaccharides. Mosaic distributions of many of these local regulators across *Bifidobacterium* species correlate with distribution of corresponding catabolic pathways. In contrast, the maltose, galactose, sucrose, and fructose regulons, as well as a novel global LacI-family regulator that is predicted to control the central carbohydrate metabolism and arabinose catabolism genes, are universally present in all 10 studied bifidobacteria. A novel group of TetR-family regulators presumably controls the glucoside and galactoside utilization pathways. Paralogs of the ribose repressor RbsR control the pyrimidine nucleoside utilization genes. Multiple paralogs of the maltose regulator MalR co-regulate large sets of genes involved in maltodextrin utilization. The inferred metabolic regulons provide new insights on diverse carbohydrate utilization networks in bifidobacteria that can be employed in metabolic modeling, phenotype prediction and the rational development of novel prebiotics.

## Introduction

Probiotics are live microorganisms which when administrated in adequate amounts confer a health benefit on the host (Hill et al., 2014). The history of probiotic concept goes back to 1908 when Metchnikoff described the positive effects of some fermented foods on human health (Metchnikoff and Schlesinger, 1908). Numerous subsequent studies confirmed this observation. The wide range of positive effects elicited by (consumption of) probiotic strains was established: protection against infectious diseases, reduction of allergic disease symptoms, immune modulation, alleviation of acute gastro-enteritis, reduction of cholesterol levels, reduction of lactose intolerance, reduction of intestinal inflammations, and production of vitamins and acetate (Goldin, 1998; Ouwehand et al., 2002; Stanton et al., 2005; Bezirtzoglou and Stavropoulou, 2011).

Prominent among the commercialized probiotic organisms are members of the genus *Bifidobacterium* (Turroni et al., 2011), which represent anaerobic, saccharolytic, high GC-content, non-sporulating Gram-positive bacteria from the Actinobacteria phylum (Ventura et al., 2007b; Pokusaeva et al., 2011a). Members of the *Bifidobacterium* genus are typically inhabitants of the animal (insects, birds, and mammals, including humans) gastrointestinal tract (GIT). Some representatives of this genus, such as *B. dentium* and *B. catenulatum*, are characterized by a cosmopolitan lifestyle as they are found in diverse ecological niches (Lamendella et al., 2008). Bifidobacteria represent one of the dominant bacterial residents in infant gut, although their relative contribution to the GIT community decreases following weaning and subsequent aging (Lee and O'sullivan, 2010; Pokusaeva et al., 2011a). Although a few cases of *Bifidobacterium* species bacteremia have been reported (Bertelli et al., 2015; Weber et al., 2015), all these cases concerned people with some health issues such as bacteremia in preterm infants and diabetes mellitus and obesity in another elderly person. These cases call for caution only when using bifidobacteria as probiotics in vulnerable patients.

Due to their positive influence on human health, some strains of *Bifidobacterium animalis* subsp. *lactis* and *Bifidobacterium longum* are commercially used as probiotics and are included in functional foods (Stanton et al., 2005; Russell et al., 2011). The common way to promote the growth of GIT commensals is to use specific dietary polysaccharides, or prebiotics, that can serve as nutrients and selectively stimulate the growth and (metabolic) activity of some GIT inhabitants (Guarner and Malagelada, 2003; Vaughan et al., 2005; Turroni et al., 2011; Bindels et al., 2015; Rastall and Gibson, 2015). Thus, understanding the metabolic pathways and underlying regulatory networks of carbohydrate utilization in bifidobacteria may provide novel ways to improve human health.

Bifidobacteria are saccharolytic organisms; their success in competition for resources in GIT microbial community depends on their ability to utilize the wide range of host- and diet-derived carbohydrates polysaccharides. High abundance of genes that are predicted to encode carbohydrate-utilizing enzymes and transporters in sequenced bifidobacterial genomes reflects their adaptation to GIT environment (Ventura et al., 2007a; Milani et al., 2014, 2015; Turroni et al., 2015). These large sets of sugar catabolic pathways require complex regulatory networks for coordination of gene expression in response to substrate availability. Carbohydrate utilization pathways in bacteria are often controlled by DNA-binding transcriptional factors (TFs) that act as repressors or activators, and induce gene expression on appearance of a sugar effector (Cohn and Horibata, 1959). A TF regulon is a group of genes and operons controlled by a common TF. Sugar-responsive TF regulons often include genes encoding enzymes and transporters from a common catabolic pathway. Despite recent characterization of several specific carbohydrate catabolic pathways and regulons in bifidobacteria (Trindade et al., 2003; Gilad et al., 2010; Pokusaeva et al., 2010, 2011b; O'connell Motherway et al., 2011; De Bruyn et al., 2013; O'connell et al., 2014; Egan et al., 2015), transcriptional regulons for many other catabolic pathways and genes are still poorly understood.

Previously, we have established a subsystems-based comparative genomics approach that was utilized for combined reconstruction of carbohydrate metabolic pathways and associated TF regulons in several diverse lineages of Bacteria including *Shewanella* (Rodionov et al., 2010), *Staphylococcus* (Ravcheev et al., 2011), *Bacillus* (Leyn et al., 2013), *Streptococcus* (Ravcheev et al., 2013a), *Bacteroides* (Ravcheev et al., 2013b), and *Thermotoga* (Rodionov et al., 2013). These and other studies have revealed substantial diversity of regulatory and metabolic networks involved in sugar catabolism. Not only different bacterial taxa utilize diverse regulatory mechanisms and metabolic pathway for utilization of a particular carbohydrate but also the substantial regulatory and metabolic network diversity was observed within some of the above lineages. In this work, we utilized a similar bioinformatics approach to investigate the regulatory network of carbohydrate metabolism in the taxonomic group of bifidobacteria. Our results predict multiple novel TFs for local control of specific carbohydrate catabolic pathways and one global regulator that co-regulates central carbon metabolism and arabinose utilization genes. Overall, we report identification of TF-binding site (TFBS) motifs and reconstruction of regulons for 64 orthologous groups of TFs in ten *Bifidobacterium* genomes.

## Materials and methods

For comparative analysis, we excluded the genomes of closely-related strains and selected 10 completely sequenced genomes of *Bifidobacterium* spp. (Table 1). The selected genomes were downloaded from Genbank (Benson et al., 2015). To define a starting set of TFs presumably regulating carbohydrate metabolism genes, we have used the protein domain search tool implemented in the Pfam database (Finn et al., 2014) and searched the selected genomes for genes containing the following domains from six known sugar-specific TF families: LacI (PF00356 and PF00532), ROK (PF00480), DeoR (PF00455), RpiR (PF01418 and PF01380), SorC (PF04198), GntR (PF00392), and AraC (PF02311). The latter two families are known to contain TFs with diverse effector ligand specificities and corresponding target metabolic pathways. Therefore, we selected for further analysis only those AraC- and GntR-family regulators that are encoded by genes located in the genomic neighborhood with prospective sugar utilization genes (Table 1). The TetR-family regulators that were previously unknown to control sugar catabolic genes have also been included in the analysis based on their genomic co-localization with the glucoside/galactoside utilization genes (Table 1). For each TF family, a phylogenetic tree of regulators from 10 analyzed genomes was constructed using PhyML (Guindon et al., 2010). We further defined orthologous groups of TFs. A group of TFs was marked as a group of orthologs if it: (i) forms a mono- or paraphyletic branch of the phylogenetic tree; (ii) has a conserved genomic context; and (iii) has highly similar TFBS motifs. The identified orthologous groups of TFs are fully described in Figure S1 and Table S1.

**Table 1:** **Distribution of TFs controlling carbohydrate metabolism in bifidobacteria**.

**Genome**	**Protein families of sugar catabolic TFs**								**Total**	
	**LacI**	**ROK**	**RpiR**	**DeoR/SorC**	**BglG**	**TetR^a^**	**AraC^a^**	**GntR^a^**	**TFs**	**CUG^b^,%**
*B. adolescentis*	18	4	0	1	1	6(10)	2(4)	1(10)	33	11.6
*B. angulatum*	19	4	0	1	1	2 (9)	1(2)	0(8)	28	11.2
*B. animalis*	11	4	0	1	0	2(15)	0(1)	0(18)	18	10
*B. bifidum*	11	4	1	2	1	0(6)	0(2)	2(11)	21	9.3
*B. breve* DSM 20213	23	7	1	1	1	2(9)	2(3)	1(12)	38	13
*B. breve* UCC2003	30	6	1	1	1	2(7)	0(1)	1(12)	42	14.1
*B. dentium*	34	6	0	1	1	7(16)	2(6)	3(13)	54	15.5
*B. gallicum*	7	3	0	1	0	0(3)	0(0)	1(9)	12	9.2
*B. longum* NCC2705	21	6	1	1	1	1(6)	0(0)	0(9)	31	11.6
*B. longum* ATCC 15697	20	5	1	1	1	1(8)	1(2)	1(12)	31	11.8

a *Total number of TFs from the TetR, AraC, and GntR families per genome is shown in parenthesis*.

b *Last column contains percent of Carbohydrate Utilization Genes (CUG) according to the IMG database*.

For identification of TFBSs and regulon reconstruction, we used the previously established comparative-genomics approach (Rodionov, 2007) implemented in the RegPredict Web server (Novichkov et al., 2010). The approach includes a number of consecutive steps: (i) inference of a TFBS motif, (ii) construction of nucleotide positional weight matrices (PWMs) for TFBSs, and (iii) genome-wide identification of additional TFBSs in all studied genomes containing a TF ortholog. A given TF frequently controls genes that are located in close proximity of the gene encoding that TF, and for this reason we defined sets of potential TF-regulated genes via genomic context analysis of a TF gene and its orthologs using genome browsers implemented in MicrobesOnline (Dehal et al., 2010) and IMG (Markowitz et al., 2014).

For TFBS motif inference, we collected upstream regions of potential TF-regulated genes in each studied genome containing a TF ortholog and searched for putative DNA motifs with palindromic symmetry using the Discover Profile tool in RegPredict (Novichkov et al., 2010). Each inferred motif was validated via the phylogenetic footprinting approach as previously described (Ravcheev et al., 2013b). Multiple DNA sequence alignments of orthologous upstream regions were constructed by ClustalW2 (Larkin et al., 2007). For each identified TFBS motif, a specific nucleotide PWM was constructed and used to search additional TFBSs and regulon members in the analyzed genomes possessing a TF ortholog. For this purpose, we used the Run Profile tool implemented in RegPredict. Upstream gene regions from −350 to +50 nucleotides relative to the presumed translation start were scanned. The *B. breve* UCC2003 genome, which is not available in RegPredict, was scanned using the Genome Explorer tool using a similar PWM-based approach and search parameters (Mironov et al., 2000). The TFBS score thresholds for site searches were usually selected 10% below the lowest site score in the training set. Weaker sites (with scores 10% less than the threshold) were also taken into account if (i) their positions were similar to positions of stronger sites (with score above the threshold) located upstream of orthologous genes and (ii) there were no competing candidate TFBSs with higher scores in the same intergenic region. To avoid false positive predictions, we filtered candidate TFBSs using consistency-check approach. New candidate members were attributed to the regulon if they were preceded by candidate TFBSs in several related genomes. The reconstructed regulons were extended to include all genes in putative operons. Genes were considered to belong to an operon if they were transcribed in the same direction, with intergenic distances not exceeding 200 nt, and when such organization persisted in several genomes. DNA motif sequence logos were drawn by WebLogo (Crooks et al., 2004).

All reconstructed regulons including TFs, TFBSs, and TF-regulated genes (except those for *B. breve* UCC2003) are captured and displayed in the RegPrecise database (Novichkov et al., 2013) and are freely available at: http://regprecise.lbl.gov/RegPrecise/collection_tax.jsp?collection_id=168. The detailed regulon reconstructions including those for *B. breve* UCC2003 are also available in Table S2.

Functional annotation of genes in the identified regulons and reconstruction of associated carbohydrate utilization pathways was performed using a combination of comparative genomics techniques (Rodionov et al., 2010) and the previous metabolic reconstructions in *Bifidobacterium* spp. extracted from the literature. Functional gene annotations were uploaded from the SEED (Overbeek et al., 2014) and UniProt (Uniprot, 2015) databases. Additionally, the closely-related gene orthologs with experimentally determined function were identified via BLAST searches in UniProtKB/SwissProt (Altschul et al., 1997). The knowledge of specificity for at least one gene in a regulon may allow predicting the specificity of the entire co-regulated pathway. For some enzymes with yet unknown substrate specificity, the protein domain analysis in Pfam allowed us to propose the general class of catalyzed biochemical reaction. Glycosyl hydrolases, that are usually the first step of a catabolic pathway, were classified and annotated using the CAZy database (Lombard et al., 2014). Prediction of TF effector and functional role was based on the analysis of available experimental data including the previously published transcriptomics data and analysis of functional content of the reconstructed regulons.

## Results and discussion

### Repertoire of carbohydrate-related TFs in *bifidobacterial* genomes

To estimate the scale and diversity of transcriptional regulators for carbohydrate utilization pathways in bifidobacteria, we collected all genes encoding TFs from the LacI, ROK, DeoR, RpiR, SorC, AraC, BglG, and GntR protein families and analyzed their genomic context in 10 *Bifidobacterium* genomes (Table 1). From the previous large-scale genomic reconstruction of regulons in bacteria, it was known that regulators from these TF families are most commonly involved in the control of carbohydrate utilization pathways (Rodionov et al., 2010, 2013; Ravcheev et al., 2011, 2013a; Leyn et al., 2013). According to the RegPrecise database (Novichkov et al., 2013), the majority of known regulators in the LacI, ROK, BglG, DeoR, SorC, and RpiR families control carbohydrate metabolism. However, TFs from the GntR and AraC families can be involved in a wide range of metabolic pathways including carbohydrate metabolism. Thus, we selected only those GntR- and AraC-family TF-encoding genes that are co-localized with genes that are predicted to be involved in the carbohydrate metabolism. TFs from the TetR family are involved in regulation of antibiotic resistance, cell-to-cell signaling and metabolism of different compounds (Cuthbertson and Nodwell, 2013). Here we found a novel group of TetR-family TFs that potentially control sugar catabolic pathways (see below); therefore we also analyzed the genomic distribution of TetR-family regulators and selected a subset of such regulators that are presumably associated with sugar metabolism.

As a result, we identified 308 potential carbohydrate-specific TFs in 10 analyzed genomes of bifidobacteria (Table 1). The total number of putative carbohydrate metabolism-associated TFs per genome varied from 12 in *B. gallicum* to 54 in *B. dentium*. Most of these regulators belong to two large TF protein families, LacI and ROK, while sugar-specific TFs from other families are less common in bifidobacteria. The LacI family constitutes 63% of the total number of identified carbohydrate-related TFs (from 7 to 34 TFs per genome), 16 and 7% of TFs belong to the ROK and TetR families, respectively, while the remaining TFs are from the AraC, BglG, DeoR, GntR, RpiR, and SorC families.

By analyzing the phylogenetic trees and the genomic context of selected regulators in each TF family, we have marked out orthologous groups of TFs (Figure S1 and Table S1). As a result, the entire set of 194 LacI-family TFs was categorized into 34 groups of two or more orthologs and 22 singleton regulators that lack orthologs in the studied genomes. In the ROK family, 47 TFs were found to be members of 8 orthologous groups, while the remaining two regulators are singleton TFs. Novel sugar utilization-related regulators from the TetR family (23 TFs) were broken into six groups of orthologs and two singleton TFs.

Overall, orthologs of the predicted sugar regulators are unevenly distributed among the 10 analyzed genomes. The most conserved TFs in Bifidobacteria that are present in all 10 genomes include the DeoR-family regulator GlcR, the LacI-family regulators CscR, MalR, and AraQ, and the ROK-family regulators NagR and BLA_0357.

### Genomic reconstruction of TF regulons

To infer transcriptional regulons for the repertoire of sugar-related TFs identified in the *Bifidobacterium* genomes, we utilized the comparative genomic approach (see Section Materials and Methods for details). This approach resulted in the identification of TFBS motifs and regulon reconstruction for 48 out of the 55 orthologous groups of TFs and for 16 out of the 33 singleton regulators (Table 2). The majority of TFs with reconstructed regulons show mosaic distribution in the studied genomes. Six TFs including the predicted regulators of sucrose, maltose, fructose and galactose utilization pathways and the predicted global regulator AraQ for central carbohydrate metabolism (see below) were identified in all studied *Bifidobacterium* genomes. For each reconstructed regulon, detailed information about orthologous TFs, their cognate TFBSs and regulated genes/operons is available in Table S2. The reconstructed regulons in 10 analyzed genomes include 268 TFs, 755 TFBSs, and more than 600 regulated operons that constitute more than 1000 genes (Table S3).

**Table 2:** **Composition of orthologous groups of sugar catabolic TFs with reconstructed regulons in 10 bifidobacterial genomes**.

**TF regulon^a^**	**Analyzed genome of *Bifidobacteria*^b^**										**Catabolic pathway^c^**
	**1**	**2**	**3**	**4**	**5**	**6**	**7**	**8**	**9**	**10**	
AbfR			+		+				++		Arabinose oligosaccharides
AouR			+				+		+		Arabinose oligosaccharides
BBNG_01789				+							?
Bbr_0019						+					?
BDP_1267					+		+				?
BDP_2071							+		+		?
BDP_2100					+		+				?
BDP_2111					+	+	+				?
BDP_2131		+					++				?
BfrR	+	+								+	Fructooligosaccharides
BgaR		+	+	+	+	+	+		+		Beta-galactosides
BglR	+	+			+	+	+				Beta-glucosides
BgrT1	+	+			+		+			+	Beta-glucosides
BgrT2	+	+			+	+	+		+		Beta-glucosides
BgrT3	+		+				+				Beta-galactosides
BgrT4	+						++				Beta-glucosides
BgrT5	+						+				Beta-glucosides
BgrT6	+										Beta-galactosides
BgrT7							+				Beta-galactosides
BgrT8			+			+					Beta-glucosides
BIFBRE_03467					+	+					?
BIFBRE_03542					+						?
BL0176	+						+		+		?
BL0185	+	+					+		+		?
BL0610									+		?
BLA_0143			+				+				?
BLA_0357	+	+	+	+	+	+	+	+	+	+	?
Blon_0374										+	?
Blon_2415										+	?
CldR^*^						+	+				*Cellodextrin*
CscR	+	+	+	+	+	+	+	+	+	+	Sucrose
FruR	+	+	+	+	+	+	+	+	+	+	Fructose
FucR					+	+				+	Fucose
GalR	+	+	+	+	+	+	+	+	+	+	*Galactose*
GalR2		+									Galactose; lactose
GlcR	+	+		+	+	+	+		+	+	Glucose
GlxR									+		Glycerate
GntR							+				Gluconate
GosR (or GalR)^*^	+	+		+	+	+	+		+	+	*Galactan*
HxlR		+					+				Hexulose
LacR	++	+	+	+	++	++	+		+	++	Lactose
MalR/MalR2/MalR3	++	++	++	+	+++	++++	++	++	++	++	Maltose, maltodextrin
MalR4							+		+		Maltose, maltodextrin
MalR5	++	+				+	+				Maltose, maltodextrin
MelR1^*^						+					*Melezitose*
MelR2^*^						+					?
MsmR	+	+					+			+	?
MsmR1					+	+	+		+	+	Alpha-galactosides
NagR				++	+	+			+	+	*Galacto-N-biose, lacto-N-biose*
NanR^*^					+	+		+		+	*Sialic acids*
PtsR	+	+		+	+	+	+	+		+	(General PTS components)
RafR^*^	+	+	+		+	+	+		+	+	*Raffinose*
RbsR							+				Ribose
RbsR2 (or RbsR)^*^					+	+			+	+	*Ribose*; (ribonucleosides)
RbsR3	+	+	+				+++	+			(Ribonucleosides)
RbtR	+				+						Ribitol; xylitol
ScrR^*^	+	+	+	+	++	++	+		++	+	*Sucrose*
SgaR				+							L-xylulose
SgaR2				+							Ascorbate
XosR	+	+					+	+			Xylo-oligosacharides
XylR	+	+	+				+	+	+	+	Xylose
AraQ	+	+	+	+	+	+	+	+	+	+	(Central carbohydrate metabolism); arabinose

a *Experimentally studied TFs are marked with asterisk; alternative TF names used in previous studies are given in parentheses*.

b *Numbered columns represent the studied genomes of Bifidobacteria: 1, B. adolescentis ATCC 15703; 2, B. angulatum DSM 20098; 3, B. animalis subsp. lactis AD011; 4, B. bifidum NCIMB 41171; 5, B. breve DSM 20213; 6, B. breve UCC2003; 7, B. dentium Bd1; 8, B. gallicum DSM 20093; 9, B. longum NCC2705; 10, B. longum subsp. infantis ATCC 15697. Multiple ‘+’ signs represent multiple paralogous TFs. All locus tags for respective TFs are included in Table S1*.

c *Predicted substrates of TF-regulated carbohydrate utilization pathways are listed; other pathways that are not specific to a particular carbohydrate are given in parentheses. Previously characterized carbohydrate catabolic pathways are in italic; the respective literature references are given in Table S1*.

Among the analyzed TFs with reconstructed regulons there are eight regulators that were previously experimentally characterized in *B. breve* UCC2003 (Table 2). These include regulators for utilization of cellodextrin (CldR), galactan (GalR that we renamed here to GosR in order to avoid the name conflict with the DeoR-family regulator of galactose metabolism), melezitose and possibly related sugars (MelR1, MelR2), raffinose (RafR), sialic acids (NanR), sucrose (ScrR), and ribose (RbsR). The reconstructed TF regulons (their gene content, assigned function, and TFBSs) as deduced from our bioinformatics-based discovery approach are consistent with the previous experimental data (Trindade et al., 2003; Pokusaeva et al., 2010, 2011b; O'connell Motherway et al., 2011; O'connell et al., 2014; Egan et al., 2015).

### Functional content of reconstructed regulons

**Figure 1:**
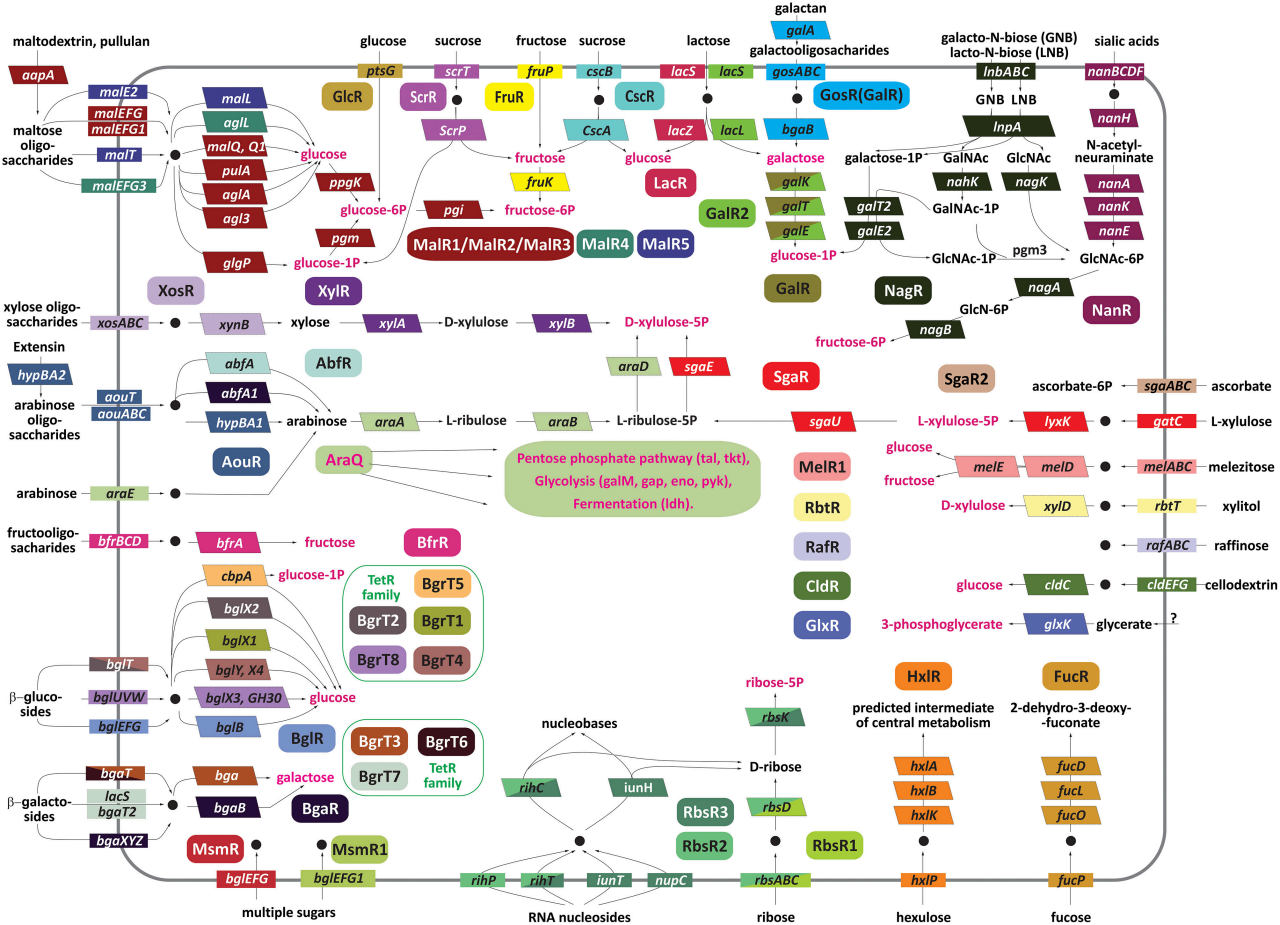
**Carbohydrate utilization pathways involved in the reconstructed transcriptional regulatory network in bifidobacterial genomes**. The regulators are denoted by ovals; transporters are shown by rectangles, and enzymes are shown by bent rectangles. The genes regulated by the same TF are shown by matching background colors. The genes that are regulated by two different regulators are colored by both colors in diagonal segments. Common intermediates of the central carbohydrate metabolism are shown in red. Note that none of the individual *Bifidobacterium* species contain all of the shown pathways. The utilized compounds transported into the cell are depicted by black circles.

To predict possible biological functions and molecular effectors of the studied TFs, we analyzed the predicted functional gene content for each reconstructed regulon. Metabolic reconstruction of the respective biochemical pathways and prediction of functions of co-regulated genes were performed using the analysis of conserved chromosomal gene clusters in genomic databases. In this manner we were able to predict metabolic pathways and/or biological functions for 46 out of 64 orthologous groups or singleton TFs (Table 2). For the remaining 18 groups of TFs, a specific sugar catabolic pathway remained unknown. Most of the studied TFs have local regulons that include 1–5 candidate TFBSs located in the promoter regions of operons that co-localize with a TF gene (Table S2). Genes from these local TFs regulons usually encode enzymes from a particular sugar utilization pathway (Figure 1).

In spite of diversity of biochemical reactions making up different carbohydrate utilization pathways, most of them are performed by a narrow repertoire of enzymatic activities including oxidoreductases, kinases, aldolases, hydrolases, and isomerases. Thirty five reconstructed regulons include glycosyl hydrolases from at least 20 different families (according to the CAZy database), suggesting their involvement in utilization of di-, oligo- and/or polysaccharides (Table S4). The analyzed glycosyl hydrolases from sugar utilization regulons were predicted to cleave glycoside bonds in certain disaccharides (sucrose, lactose, cellobiose), a wide range of oligosaccharides (β-galactosides, β-glucosides, fructo-, arabino-, galacto-, malto-oligosaccharides), and some polysaccharides such as maltodextrin and galactan (Figure 1).

In addition to enzymes, 48 regulons include one or multiple carbohydrate uptake transporters. Most of these transporters belong to the ATP-binding cassette (ABC) and Major Facilitator Superfamily (MFS) families. In addition, the glucose-specific phosphotransferase (PTS) system PtsG was found to belong to the GlcR regulon, whereas the *ptsI* and *hpr* genes encoding the general components of PTS are predicted to be regulated by the LacI-family regulator PtsR. However, some regulons such as the GlxR (glycerate) and GalR (galactose) regulons lack transporters and possess only catabolic enzymes. In contrast, certain regulons containing sugar uptake transporters (e.g., SgaR2, MsmR1, MsmR2, RafR) do not include the complete catabolic pathway, suggesting the respective catabolic enzymes are shared with other regulons or not regulated.

The reconstructed regulatory network in 10 *Bifidobacterium* species covers the utilization pathways for a variety of carbohydrate substrates (Figure 1). Some of these pathways are controlled by more than one TF. For instance, we reconstructed regulons for three orthologous groups of TFs for maltose and maltodextrin utilization pathway, eight TF groups for β-galactoside and β-glucoside utilization pathways, three TF groups for ribose and/or pyrimidine nucleoside utilization. The content of these multi-TF regulons is described in the following sections. The observed redundancy in sugar-specific TFs is explained by (i) non-orthologous replacements of TFs for the same pathway in different genomes and (ii) existence of alternative pathway variants and multiple paralogs regulated by different TFs in the same genome. For example, there are two alternative pathways for sucrose utilization by bifidobacteria involving sucrose hydrolase CscA and sucrose phosphorylase ScrP that are regulated by CscR and ScrR, respectively. The galactose catabolic pathway genes are co-regulated by the DeoR-family regulator GalR in 9 out of 10 studied genomes; however, in the genome of *B. angulatum*, these genes are co-regulated with the lactose utilization genes by another TF from the LacI family, named GalR2. In another case, two catabolic pathways for lacto-N-biose/galacto-N-biose and sialic acids that share their final enzymatic steps (NagA and NagB, see Figure 1) have diversified regulatory strategies: the sialic acid utilization genes *nanBCDFA-nagB1-nanH* are under control of the N-acetylneuraminate-responsive repressor NanR (Egan et al., 2015), whereas the lacto-N-biose/galacto-N-biose utilization genes *lnbABC-lnpA-nahK-galT2-galE2* and *nagK* are predicted to be co-regulated with the *nagA-nagB* genes via the putative N-acetylglucosamine-6-phosphate-responsive regulator NagR from the ROK family.

### Co-evolution of ribose and pyrimidine nucleoside regulons

In *Bifidobacterium breve* UCC2003, the ribose utilization genes are organized into the *rbsACBDK* operon, which encode components of ribose ABC transporter, ribose mutarotase, and ribokinase, respectively. The D-ribose-responsive repressor from the LacI family, which is encoded by the upstream gene *rbsR*, controls the *rbs* operon expression (Pokusaeva et al., 2010). We identified 11 *rbsR* homologs associated with the *rbs* genes that constitute three orthologous groups of regulators (RbsR1, RbsR2, and RbsR3) and reconstructed their respective transcriptional regulons in the 10 studied *Bifidobacterium* genomes. *B. dentium* encodes RbsR1, which controls the *rbsACBD* operon; however, the predicted DNA binding motifs of RbsR1 and the *B. breve* UCC2003 RbsR regulator are substantially different, thus we renamed the latter regulator as RbsR2. Closely-related orthologs of RbsR2 were identified in *B. breve* DSM 20213 and two strains of *B. longum*, however, their reconstructed regulons do not include the ribose utilization genes. Instead, the RbsR2 regulons in these three genomes contain ribokinase *rbsK*, non-specific ribonucleoside hydrolase *rihC*, and predicted ribonucleoside transporters *rihP* and *rihT*. The RbsR3 regulators from *B. angulatum, B. dentium, B. adolescentis, B. animalis*, and *B. gallicum* form a standalone branch on the phylogenetic tree of the LacI family (Figure S1A). However, both RbsR2 and RbsR3 regulators are characterized by similar DNA binding motifs with a common consensus TGATAAAACGTTTTATCA (Table S2). The inferred RbsR3 regulons include non-specific ribonucleoside hydrolase *rihC* and ribonucleoside transporters (*rihT* and *nupC*), and ribokinase *rbsK*. In addition, *B. dentium* has a second paralog of RbsR3 that presumably controls inosine-uridine preferring nucleoside hydrolase *iunH* and predicted inosine-uridine transporter *iunT*. Thus, we propose that several ribose-specific RbsR regulons have evolved in various bifidobacterial genomes to control the utilization of RNA-derived nucleosides (Figure 1).

### Regulation of maltose and maltodextrin utilization genes by paralogous LacI-family TFs

**Figure 2:**
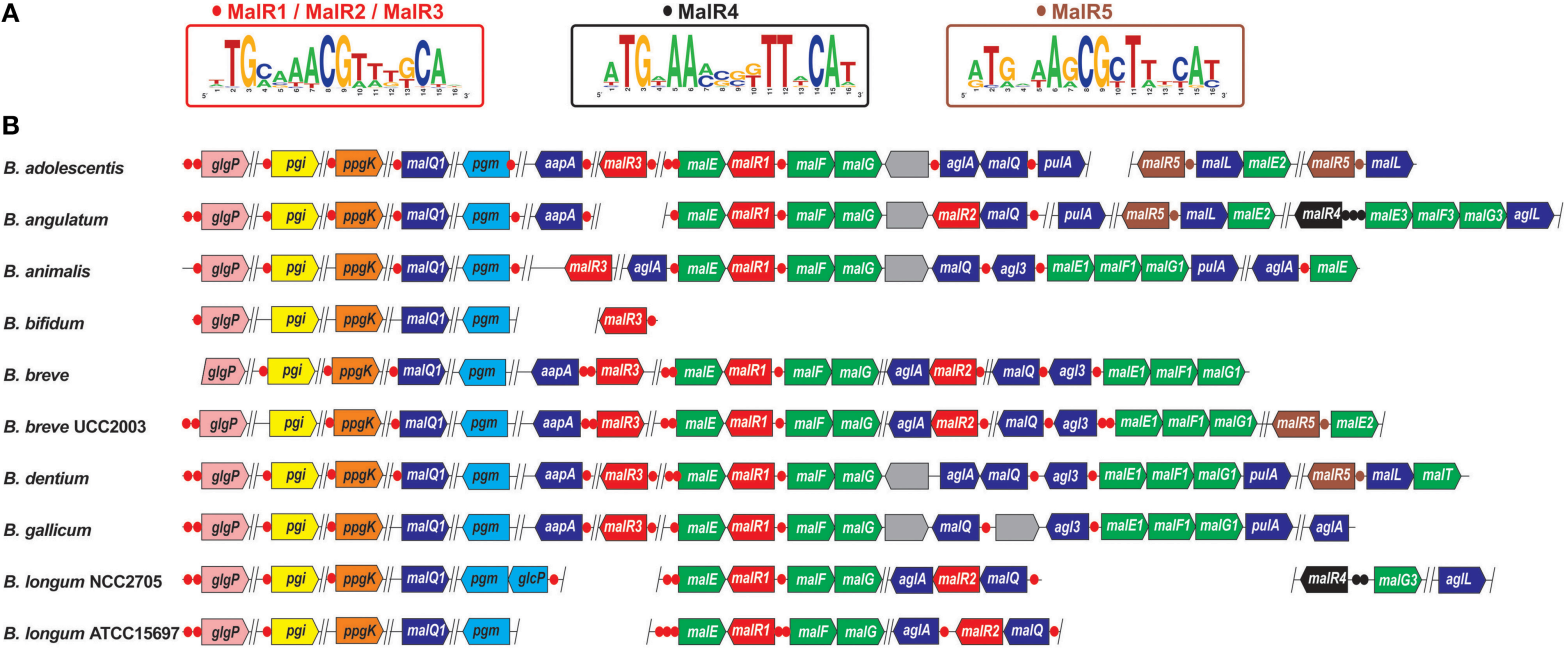
**Genomic organization of LacI-family regulons for maltose and maltodextrin utilization genes in bifidobacteria. (A)** Consensus sequence logos for predicted DNA binding sites of five orthologous groups of MalR regulators. MalR1, MalR2, and MalR3 have identical DNA motifs. **(B)** Genomic context of MalR regulons in 10 *Bifidobacterium* genomes. Candidate regulator binding sites and genes from each regulon are shown by circles and arrows, respectively. Genes encoding MalR1/MalR2/MalR3, MalR4, and MalR5 regulators, as well as their cognate binding sites, are in red, black, and brown, respectively. Maltose/maltooligosaccharide ABC transporter genes *malEFG/maEFG1/malE2/malEFG3* are in green. Glycosyl hydrolases involved in maltodextrin utilization are in dark blue (these functional roles are listed in Table S4). Glycogen phosphorylase *glgP*, glucose-6-phosphate isomerase *pgi*, glucokinase *ppgK*, and phosphoglucomutase *pgm* are in pink, yellow, orange, and light-blue, respectively. Genes of unknown function are in gray. Vertical lines separate operons that are not adjacent on the chromosome.

The analyzed 10 *Bifidobacterium* genomes encode 29 LacI-family TFs that are predicted to control a large set of genes involved in the utilization of maltose and maltodextrins, as well as of larger α-glucosidic linkage containing polymers, such as starch, amylopectin, amylose, glycogen and pullulan (Table S1). Analysis of the corresponding phylogenetic tree shows that the maltose-specific TFs constitute five paralogous groups of regulators, named MalR1 to MalR5 (Figure S1A). Regulon reconstruction revealed that TFs from three adjacent branches share highly similar TFBS motifs and thus have a joint regulon, termed MalR1/MalR2/MalR3 (Figure 2). The *malR1, malR2*, and *malR3* genes have a mosaic distribution among the studied genomes; however if any two of them co-occur in the same genome then they are often co-localized on the chromosome. Moreover, all these regulatory genes are co-regulated by a common DNA motif with other genes from the reconstructed MalR1/MalR2/MalR3 regulon, suggesting cross-regulation between these paralogous regulatory systems. The inferred maltose/maltodextrin utilization regulon contains the wide range of genes including two paralogous copies of maltose/maltodextrin ABC transporters (*malEFG, malEFG1*), extracellular α-amylase (*aapA*, previously termed *apuB*), various cytoplasmic glycosyl hydrolases such as alpha-glucosidases (*aglA, agl3*), amylomaltases (*malQ, malQ1*), and pullunase (*pulA*), and other cytoplasmic enzymes including glycogen phosphorylase (*glgP*), glucokinase (*ppgK*), phosphoglucomutase (*pgm*), and glucose-6-phosphate isomerase (*pgi*). The latter three enzymes are involved in the central metabolism of glucose and glucose-1-phosphate that are converted to fructose-6-phosphate, and thus enter the bifid shunt glycolytic pathway (**Figure 4**). Thus, the identified MalR1/MalR2/MalR3-regulated transporters and enzymes presumably constitute the complete pathway for maltose/maltodextrin/pullulan utilization (Figure 1).

Remarkably, only two out of three typical components of ABC transporters are encoded in the identified maltose/maltodextrin transport operons in bifidobacteria: a substrate-binding protein (*malE*) and two homologous permease components (*malF, malG*), but no ATPase. We propose that a missing ATPase component is encoded by a standalone conserved gene (e.g., *BL0673* in *B. longum* NCC2705), which is not co-regulated with *malEFG* in *Bifidobacterium* genomes, and that is 63% similar to MsmK, a shared ATPase component that energizes multiple carbohydrate ABC transporters in *Streptococcus pneumoniae* (Marion et al., 2011).

The reconstructed MalR1/MalR2/MalR3 regulon is conserved in 9 out of 10 studied *Bifidobacterium* genomes, whereas *B. bifidum* lacks orthologs of most of the maltose/maltodextrin utilization genes, and the remaining MalR3 ortholog presumably regulates its own expression plus the glycogen phosphorylase *glgP*. Two other orthologous groups of maltose-specific TFs, namely MalR4 and MalR5, contain presumably local regulators that control paralogous components of maltose/maltodextrin ABC transporters (*malEFG3, malE2*), putative maltose transporter (*malT*), and two cytoplasmic maltodextrin oligosaccharide glucosidases (*aglL, malL*; Figure 2).

### Control of glucoside and galactoside utilization via novel TetR-family TFs

Bacterial regulators from the TetR family are known to control genes involved in diverse biological processes including antibiotic resistance, cell-to-cell signaling and the metabolism of various compounds (Cuthbertson and Nodwell, 2013). Here we identified a novel group of 23 TetR-family regulators in bifidobacteria that are encoded in chromosomal clusters that include genes that are predicted to be involved in carbohydrate utilization. Phylogenetic tree constructed for the identified bifidobacterial TetR-family regulators and their homologs in other taxonomic groups revealed that all regulators that are co-localized with carbohydrate utilization genes form a single large branch, named BgrT for beta-glucoside regulator from TetR family (Figure S1C). The identified BgrT regulators were mostly from the *Bifidobacterium* genus and several other lineages of Actinobacteria, as well as from two proteobacterial lineages (Rhizobiales, Pseudomonadales). In most of these lineages, the *bgrT* genes are co-localized with genes encoding various β-glucoside or β-galactoside hydrolases (e.g., *bglB, bgaB, bglX, bglY*), cellodextrin phosphorylase (*cbpA*) and predicted β-glucoside or β-galactoside transporters from the MFS (*bglT, bgaT, lacS*) and ABC (*bglUVW*) families (Figure 1).

**Figure 3:**
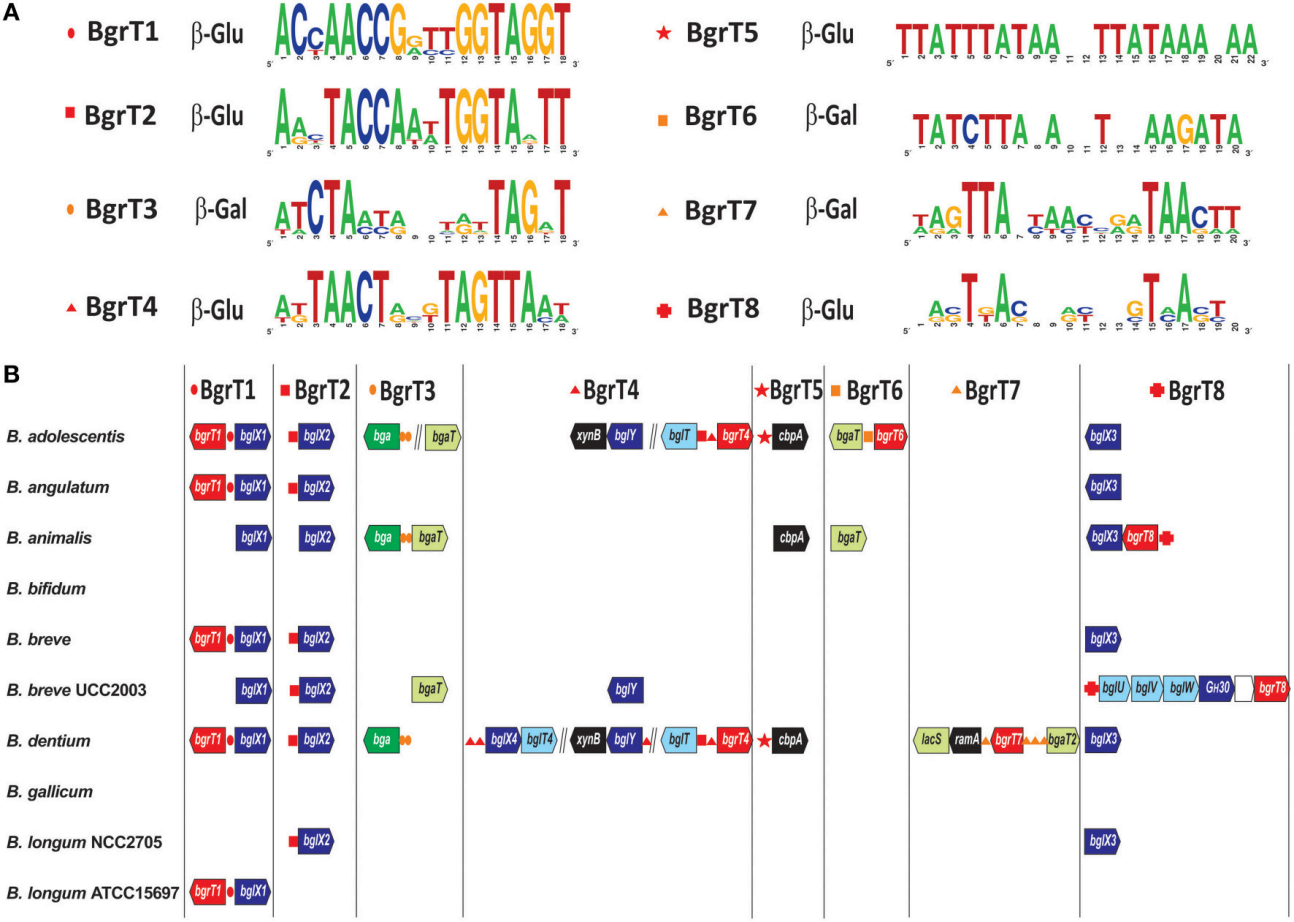
**Genomic organization of TetR-family regulons for β-glucoside and β-galactoside utilization genes in bifidobacteria. (A)** Consensus sequence logos for predicted DNA binding sites of eight orthologous groups of BgrT regulators. The predicted carbohydrate specificity of regulators toward β-glucoside (β-Glu) or β-galactoside (β-Gal) is indicated. **(B)** Genomic context of BgrT regulons in 10 *Bifidobacterium* genomes. Genes from each regulon are shown by arrows. Candidate regulator binding sites are shown by small red and orange symbols before their respective target genes. Genes encoding predicted β-glucoside and β-galactoside transporters are in light-blue and light-green, respectively. β-glucosidases and β-galactosidases are in dark blue and green, respectively. Other glycosyl hydrolases and phosphorylases are in black. BgrT regulators are in red. Vertical lines separate operons that are not adjacent on the chromosome.

For regulon reconstruction, we used the phylogenetic tree and genome context analysis to classify the 23 identified BgrT proteins from the 10 analyzed bifidobacteria into eight orthologous groups, named BgrT1-BgrT8. The obtained results allowed us to predict a unique TFBS motif, represented by a 18–22 nt palindrome, and reconstruct local regulon for each of these TF groups (Figure 3). Five reconstructed BgrT regulons contain β-glucoside utilization genes, whereas the remaining three BgrT regulons are involved in β-galactoside utilization. Most of these regulons are present in a few species. The most widespread regulons are BgrT1 (five organisms) and BgrT2 (six organisms), both of which include the adjacent β-glucosidase gene *bglX*. The largest number of *bgrT* regulators was found in *B. dentium* and *B. adolescentis*: each of these genomes contains three separate regulons for β-glucoside utilization genes and two regulons for β-galactoside utilization. Interestingly, the above two genomes have a unique regulatory cascade between BgrT2 and BgrT4, when the former TF control the divergently transcribed genes *bgrT4* and *bglT*, since their common regulatory region contains both BgrT4- and BgT2-binding sites. Thus, the β-glucoside utilization regulatory network in these two species appears to have evolved into a more complex structure compared to other studied bifidobacteria.

### A predicted global regulon for central carbohydrate metabolism genes

A novel LacI-family transcriptional regulator, named *araQ*, was identified immediately upstream of the arabinose catabolic operon *araBDA* in 6 out of 10 *Bifidobacterium* genomes. In *B. angulatum*, the *ara* operon also includes the arabinose permease *araE*. The remaining four studied genomes (namely, *B. bifidum, B. longum* subsp. *infantis* ATCC 15697, and both strains of *B. breve*) lack the arabinose utilization genes; however we observed that these four genomes apparently have preserved *araQ* orthologs. By analyzing the upstream gene regions of *ara* operons and *araQ* genes, we identified the putative AraQ-binding motif, a palindromic sequence with consensus TGTGAGCGCTCACA, that is partially similar to DNA motifs of other regulators from the LacI family (Ravcheev et al., 2014). A genomic search for additional sites using this DNA motif and their comparative genomics analysis in 10 *Bifidobacterium* genomes revealed the global AraQ regulon containing up to 19 genes per genome (Table S2).

**Figure 4:**
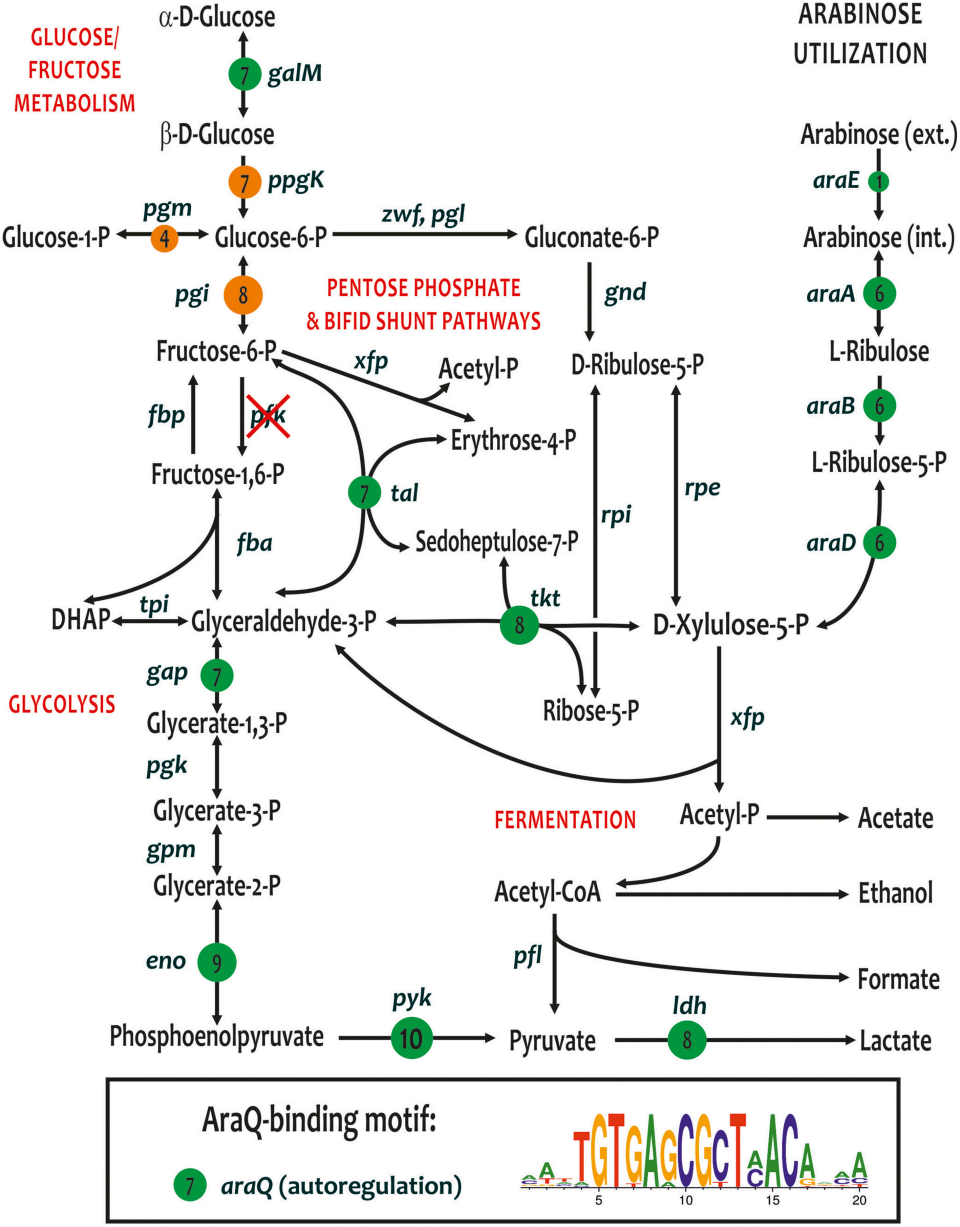
**Metabolic context of conserved core of the reconstructed AraQ regulons in the genomes of bifidobacteria**. The AraQ-regulated genes are shown by green circles. Numbers in circles show the numbers of genomes where gene is preceded by a candidate AraQ-binding site. Consensus sequence logo for the predicted AraQ-binding sites in 10 *Bifidobacterium* genomes is shown in a box insert. Additionally, the MalR-regulated genes are shown by orange circles.

In addition to the *ara* operons and *araQ* genes, the most conserved members of AraQ regulon are involved in the central glycolytic pathways including aldose-1-epimerase (*galM*), the pentose phosphate and Bifid shunt pathway enzymes (*tal, tkt*), enzymes from the lower part of glycolysis (*gap, eno, pyk*), and lactate dehydrogenase (*ldh*) (Figure 4). Interestingly, the predicted AraQ regulon does not overlap with the MalR regulon that controls several other central enzymes from the glucose/fructose metabolism (Figure 4). All studied strains of *B. breve* and *B. longum* have additional predicted AraQ regulon members involved in maltose/maltodextrin utilization, namely maltose ABC transporter (*malE*) and amylomaltase (*malQ1*), as well as 1,4-alpha-glucan branching enzyme (*glgB*), biotin ligase (*birA*), and two hypothetical proteins (*Blon_2289-90*). Thus, some *malE* and *malQ1* genes are under dual regulation of AraQ and MalR. In *B. adolescentis* and *B. dentium*, the reconstructed AraQ regulons include maltose acetyltransferase (*maa*), which is known to have broad substrate specificity being capable of acetylating many sugars. Its specific physiological role is not known but it is thought that the enzyme prevents the accumulation of free sugars to high levels through acetylation, playing a detoxifying role in the cell. Other genome-specific members of the AraQ regulons include a hypothetical glycosyl hydrolase operon in *B. adolescentis* (*PF07944-aga*) and the L-xylonate utilization operon in *B. bifidum*, which is also controlled by the local regulator SgaR.

The *araQ* gene was recently considered as one of the core genes shared by 45 *Bifidobacterium* type strains (Lugli et al., 2014; Milani et al., 2014; Sun et al., 2015). In contrast, the arabinose utilization operon *araBDA* was identified in all but seven *Bifidobacterium* type strains that are *B. bifidum, B. boum, B. breve, B. choerinum, B. longum* subsp. *infantis, B. ruminantium*, and *B. thermophilum* (data not shown). Detailed analysis of 10 *Bifidobacterium* genomes presented in this work suggests that the global control of multiple genes from the central carbohydrate metabolism is a common feature of the reconstructed AraQ regulons in all bifidobacteria (Figure 4). *Gardnerella vaginalis*, another representative of the *Bifidobacteriaceae* family, possesses an orthologous AraQ regulator (51% protein identity), which presumably controls the central carbohydrate metabolism genes (*eno, gap, pyk*); however the *ara* genes are apparently missing from this genome. Additional genomic searches outside of the *Bifidobacterium* and *Gardnerella* genera have identified distant homologs of *araQ* in several other lineages of the *Actinomycetales* (35–40% of protein identity). Most of these *araQ* homologs are co-localized with the *ara* operons that are preceded by putative AraQ-binding sites (data not shown); however, we were unable to find other distantly located AraQ sites in these *Actinomycetales* genomes. These observations suggest that a plausible scenario for the evolution of AraQ regulons. Local AraQ regulators for the arabinose utilization genes were likely to be present in a common ancestor of the *Actinomycetales*. In the common ancestor of the *Bifidobacterium* and *Gardnerella*, the AraQ regulons were further expanded to include the central carbohydrate metabolism genes. Finally, the arabinose utilization pathway genes (and their cognate AraQ-binding sites) were lost in a number of the *Bifidobacteriaceae* species, although their global AraQ regulons seem to have been preserved. The molecular effector of AraQ is unknown. A plausible hypothesis is that AraQ dissociates from its DNA sites in response to an intermediate of the central glycolytic pathway, e.g., D-xylulose-5-phosphate, which is a final product of the arabinose catabolism.

### Conclusions and future perspectives

Transcriptional regulation of carbohydrate metabolism has been previously studied in several model species from the *Bifidobacterium* genus, most notably in *B. breve* UCC2003. In this work, we applied the knowledge-driven comparative genomics to investigate transcriptional regulation of carbohydrate metabolism in bifidobacteria. The inferred sugar catabolic transcriptional regulatory network is operated by a large set of duplicated TFs that are scattered across 10 analyzed bifidobacterial genomes. Most of these TFs belong to the LacI and ROK protein families; however TFs from several other families were also identified. Interestingly, we found that the TetR family, which was not previously known to contain sugar-specific regulators, contains several novel regulators of glucoside and galactoside utilization genes in bifidobacteria. Finally, we were able to predict TF-binding site motifs and reconstruct regulons for 268 out of 308 potential carbohydrate-specific TFs that constitute 64 groups of orthologous TF regulons. Most of the analyzed regulons are local, when a TF binds one or multiple sites located in close proximity to a TF-encoding gene on the chromosome, and thus controls a relatively small set of genes that are usually involved in the same catabolic pathway. Only a small number of regulons are present in all 10 genomes; such a mosaic distribution of sugar-catabolic regulons suggests that the regulated catabolic pathways are often required for successful adaptation to particular environmental niche. In contrast, some regulatory systems, such as those controlling catabolism of sucrose, fructose, lactose, glucose, galactose, and maltose, are conserved in most of the studied genomes. The latter sugars are likely to be abundant in the habitats of the analyzed bifidobacteria and thus loss of these metabolic activities may cause a competitive disadvantage to these particular bifidobacteria.

Among novel TF regulons identified in this study, AraQ is predicted to be the first global regulon for genes involved in central carbohydrate metabolism. It is not surprising that bifidobacteria regulate the expression of genes specifying their central carbohydrate metabolic pathway. Functionally analogous (but non-orthologous) global regulators of the central carbon metabolism were previously described in other bacterial lineages including the Enterobacterial Cra/FruR and the *Shewanella* HexR regulators (Leyn et al., 2011), as well as two novel LacI-family TFs recently identified by the comparative genomics approach in α-proteobacteria (Ravcheev et al., 2014). Bifidobacteria are commonly found in the gastrointestinal tract of humans and other animals, an environment which is rich in complex carbohydrates derived both from the diet and the host epithelium cells. Thus, the ability of gut-associated microorganisms to induce the expression of genes involved in utilization of these complex carbohydrates is important to enhance their competitiveness and adaptive abilities.

The reconstructed regulatory network highlights a correlation (*R*^2^ = 0.95) between the number of carbohydrate-specific TFs and the total number of genes involved in carbohydrate metabolism in the studied genomes (Table 1). Obviously, the genomes with lower number of carbohydrate utilization genes have fewer number of carbohydrate metabolism-associated TFs. For instance, the genome of the opportunistic cariogenic pathogen *B. dentium*, was found to contain 54 sugar-specific TFs and possesses a genome with 15.5% of genes involved in carbohydrate metabolism. In contrast, the genome of *B. gallicum* is predicted to encode only 12 sugar-specific TFs, while 9.2% of its genes represent carbohydrate-metabolizing genes.

The current bioinformatics work significantly improves our understanding of carbohydrate metabolism and its regulation in bifidobacteria. Exploitation of the knowledge on the obtained regulatory network will assist in the development of novel food supplements that positively affect human health. However, a significant number of predicted regulatory interactions between novel TFs and their binding sites and target genes await future experimental validation. Systematic comparison of regulons reconstructed from genomic data with results of experimental studies (e.g., from whole-genome transcriptomics studies of TF knockout mutants) will provide a comprehensive knowledge of the carbohydrate metabolic and regulatory networks in bifidobacteria.

## Author contributions

DR conceived and designed the research project. MK and SL performed comparative genomic analysis to reconstruct TF regulons. DR provided the quality control of annotated regulons in the RegPrecise database. MK analyzed statistical properties of regulons. MK, DR, and DS wrote the manuscript. All authors read and approved the final manuscript.

### Conflict of interest statement

The authors declare that the research was conducted in the absence of any commercial or financial relationships that could be construed as a potential conflict of interest.
