# Supplementary material for: Transcriptional Regulation of Carbohydrate Utilization Pathways in the Bifidobacterium Genus
Source: Front Microbiol. 2016 Feb 9;7:120. doi: 10.3389/fmicb.2016.00120 (PMC4746261; doi:10.3389/fmicb.2016.00120)
Supplement: Supplementary file 5 [file Image1.PDF]

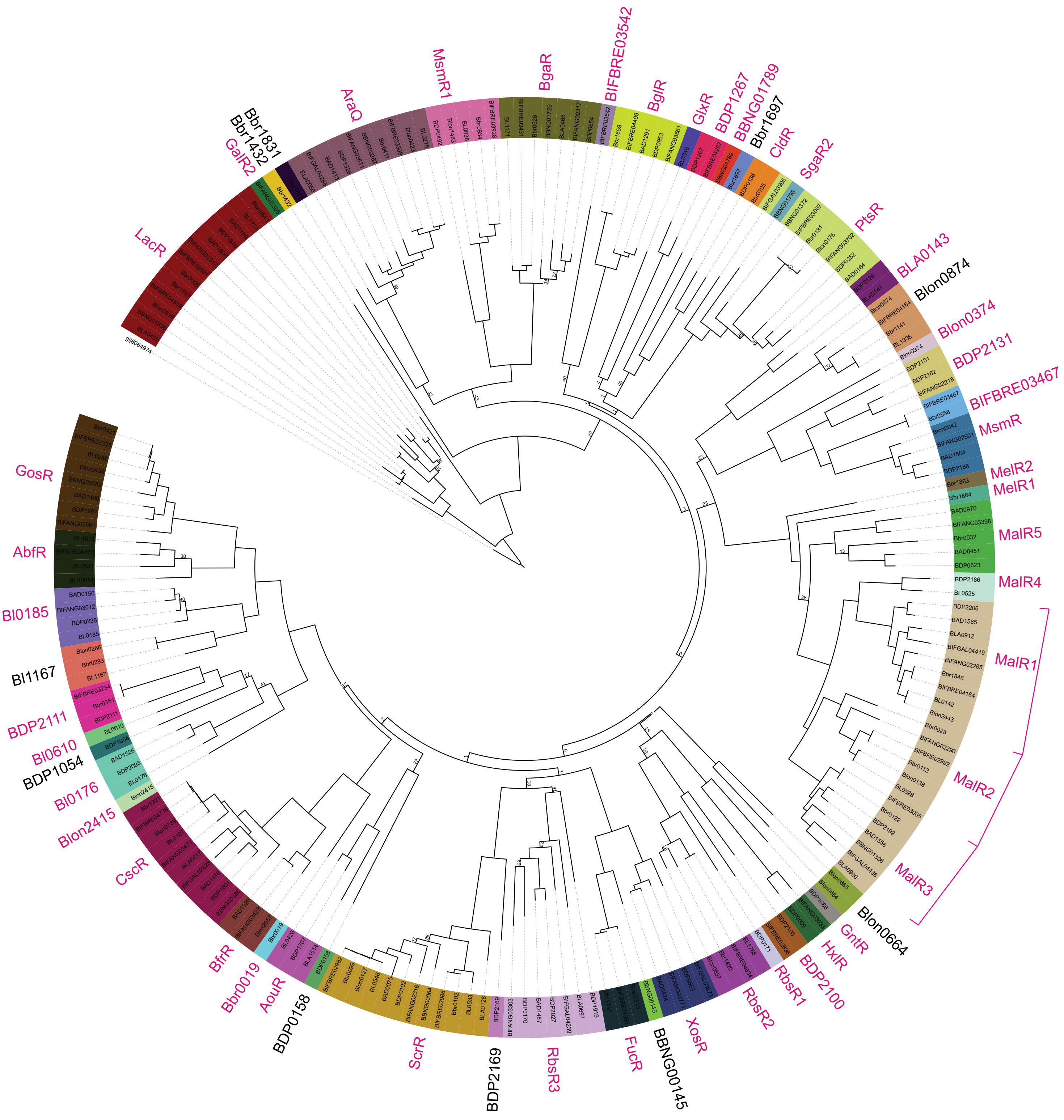

Figure S1. (A) Phylogenetic tree of the LacI-family TFs presumably involved in the control of carbohydrate utilization genes.

Tfs from the same orthologous group are highlighted by the same background color. TF orthologous group names are given in the outer circle. Groups of TFs with reconstructed regulons are shown in red.

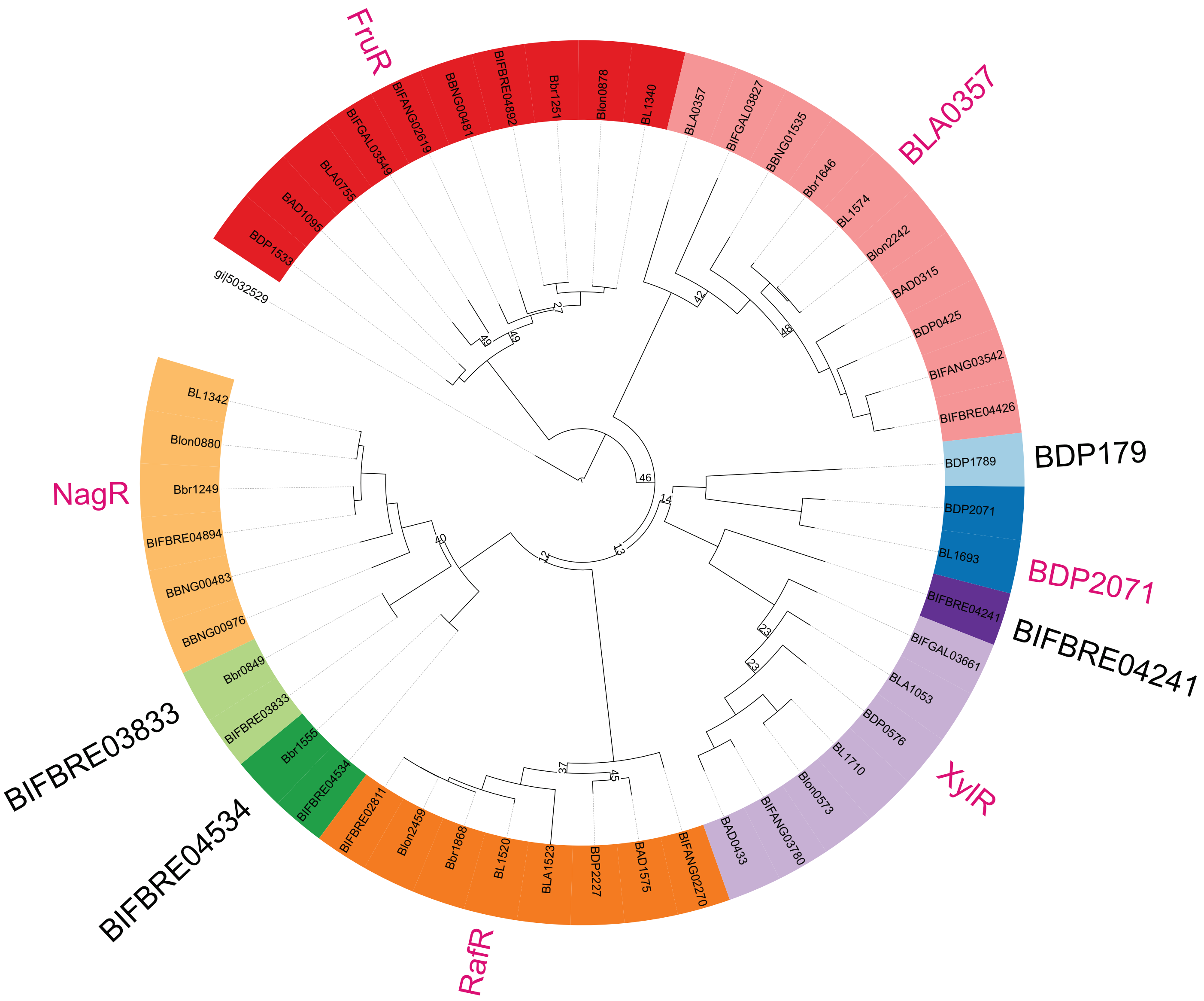

Figure S1. (B) Phylogenetic tree of the ROK-family TFs presumably involved in the control of carbohydrate utilization genes.

Tfs from the same orthologous group are highlighted by the same background color. TF orthologous group names are given in the outer circle. Groups of TFs with reconstructed regulons are shown in red.

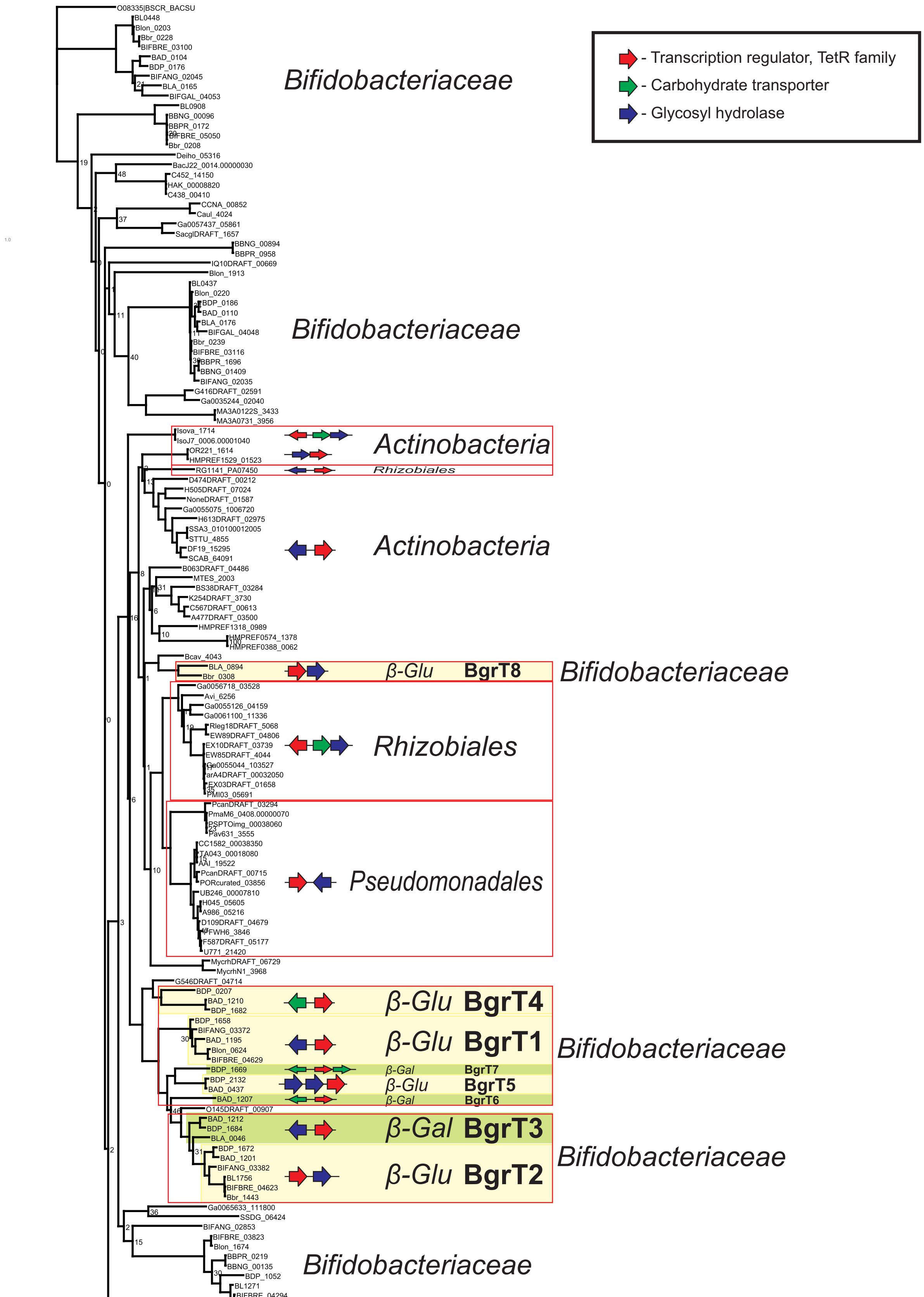

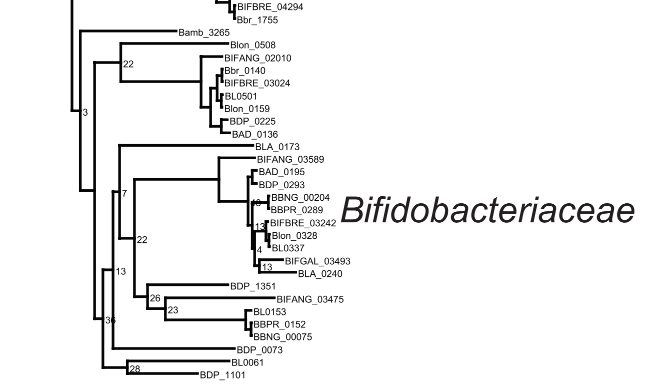

Figure S1. (C) Phylogenetic tree of the TetR-family TFs presumably involved in the control of carbohydrate utilization genes.

Tfs encoded by genes that are co-localized with genes for glycosyl hydrolases and/or carbohydrate transporters are highlighted in red boxes. Genomic context of these TetR-family regulators is shown by arrows that denote genes encoding TFs (in red), glycosyl hydrolases (in blue), and carbohydrate transporter (in green).
Eight orthologous groups of BgrT1-BgrT8 regulators with reconstructed regulons in Bifidobacteria are marked are highlighted by background color, BgrT regulons in light green and yellow are involved in utilization of beta-glucosides and beta-galactosides, respectively.
